# Supplementary material for: Modeling the shared risks of malaria and anemia in Rwanda
Source: PLoS One. 2024 Apr 22;19(4):e0298259. doi: 10.1371/journal.pone.0298259 (PMC11034660; doi:10.1371/journal.pone.0298259)
Supplement: Attachment — Submitted filename: Plagia report.pdf [file pone.0298259.s003.pdf]

review

by M Po

Submission date: 13-Aug-2023 01:03PM (UTC+0300)
Submission ID: 2145056049
File name: Malaria_PONE-D-23.pdf (705.03K)
Word count: 5025
Character count: 27669

Figure Click here to access/download;Figure;FIG1.tiff

Modeling the shared risks of malaria and anemia in Rwanda

Pacifique Karekezi[1], Jean Damascene Nzabakiriraho[2], and Ezra Gayawan[3]

[1]African Institute for Mathematical Sciences (AIMS), Kigali, Rwanda
[2]African Centre of Excellence in Data Science, University of Rwanda, Kigali, Rwanda
[3]Department of Statistics, Federal University of Technology, Akure, Nigeria

Abstract

Background: Malaria and anemia jointly account for the high morbidity and mortality among under-five children in sub-Saharan Africa including Rwanda. Notwithstanding different intervention programs and policies to address such double burden in Rwanda, the rate of infection remains a public health challenge. This study aimed at understanding the geographical variations between the joint and specific risks of both diseases in the country while quantifying the effects of some socio-demographic and climatic factors.

Methods: Using data extracted from Rwanda Demographic and Health Survey, a shared component model was conceived and inference was based on integrated nested Laplace approximation.

Results: The study findings revealed similar spatial patterns for the risk of malaria and the shared risks of both diseases, thus confirming the strong link between malaria and anaemia. The spatial patterns revealed that the risks for contracting both diseases are higher among children living in the districts of Rutsiro, Nyabihu, Rusizi, Ruhango, and Gisagara. The risks for both diseases are significantly associated with type of place of residence, sex of household head, ownership of bed net, wealth index and mother's education level. Temperature and precipitation also have substantial association with both diseases.

Conclusions: When developing malaria intervention programs and policies, it is important to take into account climatic and environmental variability in Rwanda. Also, potential intervention initiatives focusing on those living in rural areas, the lowest wealth index, children whose uneducated mothers, and high risky regions need to be reinforced.

Keywords: Shared risks; malaria; anemia; spatial analysis; Rwanda

Figure
Click here to access/download;Figure;FIG2.tiff

Introduction

Malaria and anemia are two major public health issues that have persisted in most developing countries notwithstanding the numerous intervention programmes and policies by government and donor agencies. The two diseases are endangering people's well-being and survival chances especially among under-five children and pregnant women [1, 2]. Malaria parasite is known to have substantial causative effects for the occurrences and magnitude of other diseases and leading to the death of the victim in some cases. The parasites consume iron molecules in red blood cells, leading to low functional hemoglobin concentrations. In the case of under-five children, low functional hemoglobin concentrations causes severe anemia and prevents quick recovery [3]. Iron deficiency is the most common cause of anemia, which occurs in situation where there is insufficient amount of iron in the red blood cells [4]. Malaria parasites attack the red blood cells by feeding on iron in the blood cell, causing a shortage in hemoglobin (Hb) concentration level. Epidemiological evidence has effectively demonstrates the synergy between the causal relationship of malaria and anemia. Compared to other parasites, *Plasmodium falciparum* can encroach into the red blood cell leading to acute hemolysis thereby distorting the development of the red cell leading to severe anemia [5]. Severe anemia equally worsens morbidity from malaria particularly in children [6].

In the presence of malaria, anemia is considered a health risk for under-five children, and it has been documented that it is the most causing factor of morbidity and mortality among young children in both tropics and subtropics [7]. Anemia is a condition in which the blood does not contain enough healthy red blood cells. It is caused by a shortage of red blood cells or a malfunction of existing red blood cells, which reduces the flow of oxygen in the body since red blood cells deliver oxygen to the body tissues [8]. Anemia is a severe global public health issue that disproportionately affects under-five children and pregnant women. According to the World Health Organization, 42 percent of children under the age of five and 40 percent of pregnant women are anemic worldwide [9]. Anemia among young children jeopardizes the quality of life, coupled with symptoms like increased heart rate, poor wound and tissue healing, and delayed development and growth, and consequently, leading to a poor cognitive performance and possibly leading to death [10].

Malaria and anemia put direct pressure on the already weak health care systems of most developing countries, and also directly impact individuals and households because of the loss of income and other economic consequences [2]. Most developing countries are malaria endemic areas, with approximately 300 million clinical cases and 2 million deaths recorded yearly. An estimated 229 million malaria cases worldwide were reported in 2019, with only 29 countries accounting for 95 per cent of the amount. Malaria deaths were 409,000 in 2019 and about 67% were under-five children [11]. About 93% of all malaria cases and 94% of malaria deaths reported in 2018 were from sub-Saharan Africa with under-five children accounting for more than two-thirds of all the deaths [9]. These were recorded in spite of the numerous government policies and interventions from several international development agencies.

Figure Click here to access/download;Figure;FIG3.tiff

Malaria is the second leading cause of morbidity in Rwanda, accounting for about 7.4 per cent of the total outpatient hospital visits and 4.3 percent of malaria proportional mortality [12]. Since 2012, malaria frequency has risen successively in the country from 48 per 1,000 in 2012 to 403 per 1,000 in 2016. The total number of reported malaria cases rose astronomically from 567,407 in 2012 to 4,794,778 in 2016 [13]. This indicates that malaria gains in Rwanda are fragile, and so, effective interventions need to be scaled up. Some studies have attributed the sudden increase to human behaviour, climatic change, environmental modification, and insecticide resistance [13, 14]. For instance, the incidence of malaria has been linked to altitude, with a higher prevalence in lowlands than in highlands settings, and more cases occur around May-June and NovemberDecember. In addition to favorable climate, closeness to marshlands, irrigation schemes, and cross border movement of people influence the transmission, particularly in the Southern and Eastern fringes of the country [14, 15]. As earlier argued, higher malaria risk in a given location would also imply higher risk of anemia.

Several studies have been undertaken to evaluate the incidence and determinants of malaria and anaemia among under-five children in Rwanda including their geographical distributions [1, 14, 16, 17, 18]. However, limited studies have modeled co-morbidity of the two disease, and there exists a persistent knowledge gap in their spatial overlap that specifically identify districts with high and low risks of the combined diseases. Moreover, only few studies have mapped malaria and anaemia prevalence while controlling for climatic conditions particularly temperature and rainfall in the country. It is important to understand the patterns of the spatial variations in the joint (shared) and specific risks of malaria and anaemia at small scale levels across the country, and to quantify the dynamics of climatic conditions for the two diseases as these could effective aid intervention programs that are aimed at addressing their occurrence among young children. Consequently, this study was designed to estimate the shared and specific spatial variations in malaria and anemia among children under five years of age in Rwanda using data extracted from the 2014-2015 Rwanda Demographic and Health Survey (RDHS). We adopt a shared-component modeling approach within a Bayesian framework that allows for simultaneous estimation of metrical variations as nonlinear effects, location-specific random effects that account for heterogeneity and the usual linear effects of catagorical variables. The model separates the underlying risk surface for the diseases into a shared-component common to both diseases, and disease-specific components. The shared spatial component can be conceived as a surrogate for unobserved covariates that have spatial structure common to both diseases. We considered the effects of temperature and rainfall as climatic in addition to other demographic factors on the shared risks of the two diseases. To produce approximations to the posterior marginals of all parameters of interest, we adopt the Integrated Nested Laplace Approximations (INLA).

Figure

Click here to access/download;Figure;FIG4.tiff

Methods

Data

The study relied on data extracted from the 2014-2015 RDHS. The survey, was conducted between November 2015 and April 2015 by the National Institute of Statistics of Rwanda in collaboration with the Rwanda Biomedical Center (RBC) and Rwanda Ministry of Health (MoH). The survey allows for the estimation of key indicators at the national level, and for urban and rural areas, the five provinces and each of Rwanda's 30 districts. A two-stage sample design was adopted where at the initial step sample sites (clusters) made up of enumeration areas (EAs) defined for the 2012 Rwanda Population and Housing Census (RPHC) were chosen. A total of 492 clusters were selected, with 113 in urban and 379 in rural areas. At the second stage, the households to be included in the survey were picked at random from these lists.

A total of 12,793 households were selected comprising of about twenty-six households from each EA. All women aged 15 to 49 years, who were either permanent residents or visitors who stayed in the household the night before the survey were eligible for an interview. A total of 13,564 eligible women were identified out of which 13,497 were interviewed, yielding a a response rate of 99.5 percent. Information was also elicited on children younger than five years of age in all the households. Anemia and malaria testing were executed in the sub-sample of households not selected for the male survey. After informed consent of the parent or guardian was secured, children were tested for malaria and anemia. The malaria test were done in two forms using rapid diagnostic test (RTD) and thick and thin blood smears. A drop of blood was taken by pricking the end of the finger in the case of RDTs which was frequently done at the same time as anemia testing.

Ethics Statement

The study used a secondary data obtained from the Demographic and Health Survey after approval was obtained.

Statistical analysis

Shared component model

The shared components models as described by Knorr and Best [19] allows for the estimation of the shared pattern of commonalities between two or more diseases and the ones specific to each disease. Consider a response variable y_{ijd}, taking

Figure Click here to access/download;Figure;FIG5.tiff

value of 1 if a child i from district j is suffering from disease d, and 0 otherwise, where, $i = 1,2,\cdots,3200$, $j = 1,2,\cdots,30$, and $d = 1,2$. The response y_{ijd} can be assumed to follow a Bernoulli distribution denoted as

$$y_{ijd} \sim \text{Bernouilli}(p_{ijd}), \quad (1)$$

where

$$f(y_{ijd}|p_{ijd}) = p_{ijd}^{y_{ijd}}(1 - p_{ijd})^{1-y_{ijd}} \quad (2)$$

$$= \exp[y_{ijd}\eta_{ijd} - \log(1 + \exp(\eta_{ijd}))], \quad (3)$$

and $p_{ijd} = P(y_{ijd} = 1)$ and η_{ijd}, the predictor for a child i who lives in district j, given that $\eta_{ijd} = logit(p_{ijd})$. Consequently, with the logit link function, the predictor can be linked to covariates as follows:

$$logit(p_{ijd}) = \alpha + \omega\beta + g(v_j) \cdot \sigma_d + g_d(s_j), \quad (4)$$

where α denotes the intercept common to the two diseases, β is a vector of parameters for categorical variables shared by the two diseases, ω represents a vector of all the categorical variables, $g(v_j)$, the common spatial field for all diseases, σ_d is the loadings for the common component into each disease group, which controls how disease d is affected by the shared field, and $g_d(s_j)$ is the disease-specific spatial components.

Model implementation was based on Bayesian inference through the integrated nested Laplace approximation (INLA) as proposed by [20]. The prior distribution for σ_d was log-normal with a mean of 0 and accuracy of 0.1. A weakly informative Gaussian prior $B \sim N(0,\tau_\beta I)$ with small precision τ_β on the identity matrix was considered, and used for the fixed parameters and constant term. The shared and non-shared nonlinear terms were modelled using a random walk of order two.

The intrinsic conditional auto-regressive (ICAR) prior was used to model the shared and specific spatial random components with precisions τ_v and τ_s respectively. Through the adjacency structure of the area units, spatial auto-correlation is was induced. The ICAR prior for $s = (s_1,...,s_n)^T$ is given in its simplest form as

$$\pi(s|\tau) \sim \frac{1}{Z_n(\tau)} \exp\left(-\frac{\tau}{2}\sum_{i\sim j}(s_i - s_j)^2\right), \quad (5)$$

Figure Click here to access/download;Figure;FIG6.tiff

where $i \sim j$ refers to the collection of all pairs of neighbours, with two districts considered neighbours if they share a common boundary. $Z_n(\tau)$ is the normalizing constant while τ is precision parameter. The amount of smoothness is determined by this precision parameter. We further assigned hyperpriors to the precision parameters based on penalized prior (PC prior), which is a unified prior definition for complicated models that penalizes the model's hierarchical structure. The PC prior is based on a type-2 Gumbel distribution with parameters τ and λ set to 1 and 0.01 respectively [21]. Scaling of the hyperpriors is required to ensure that all spatial components have the same degree of smoothness.

Results

Table 1 presents results of the linear effects for malaria and anemia showing the posterior means, standard deviations, and 95% credible intervals. When compared to children from rural areas, children from urban settings are less likely to develop both diseases. Children from male-headed households are less likely to have both diseases compared with those from female-headed households. whereas ownership of bed nets in households shows lower likelihood for the contracting both diseases, however, the estimates for usage of bed nets are not significant. The study findings revealed an association between wealth quintile and coexistence of the two diseases such that children from at least the poorer households are less likely to contract both diseases when compared with those from the poorest households. Children whose mothers attained primary level of education are less likely to suffer from both diseases when compared with those with no education but the result for secondary/higher education is not significant. The findings on the sex of the children show non-significant effects.

Table 1: Estimated posterior means with 95 % credible intervals for the linear effects

Parameter	Mean	sd	0.025quant	0.975quant
Place of residence				
Rural	1.000			
Urban	0.897	0.102	0.733	1.095
Sex of a household head				
Female	1.000			
Male	0.758	0.066	0.666	0.862
Bed-net ownership				
No	1.000			
Yes	0.588	0.105	0.478	0.720

Figure Click here to access/download;Figure;FIG7.tiff

Bed-nets usage				
No child uses bednet	1.000			
All use bednets	0.969	0.094	0.807	1.168
Some of them use bednets	1.153	0.133	0.887	1.497
Wealth Index				
Poorest	1.000			
Poorer	0.737	0.083	0.626	0.866
Middle	0.673	0.088	0.565	0.800
Richer	0.582	0.099	0.479	0.707
Richest	0.492	0.132	0.379	0.636
Mother's education level				
No education	1.000			
Primary	0.744	0.066	0.654	0.847
Secondary/ higher	0.887	0.119	0.702	1.118
Child-sex				
Female	1.000			
Male	0.936	0.057	0.838	1.047

The results for the spatial effects presented in Figure 1 - 3, showing the maps for the posterior means, standard deviations, and 95% credible intervals, respectively for the shared effects of malaria and anemia, specific-effects for malaria and for anemia, with the results displaying uneven distributions of the share of the diseases across the districts of Rwanda. The findings from the shared random effects (Figure 1) reveal that the highest risks of contracting malaria and anemia can be found among children from Rutsiro, Nyabihu, Ruhango, Gisagara, and Rusizi districts, while those from Rubavu, Karongi, Ngororero, Kayonza, Gatsibo, Rwamagana, Gasabo and Rulindo have moderate risks of contracting both diseases. The joint effects of Malaria and anemia seem to be negligible across other districts of the country. The standard deviation and credible intervals maps show that there is more uncertainty about the risk of malaria and anemia in various parts of the country, particularly in the districts of Nyagatare, Rubavu, Ruhango and Nyamasheke.

In terms of the specific components, the spatial distributions for malaria (Figure 2) reveal a pattern that is somewhat similar to the shared spatial pattern obtained for the two diseases. Specifically, the risks for malaria are highest among children living in

Figure Click here to access/download;Figure;FIG8.tiff

Rutsiro, Nyabihu, Ruhango, Rusizi and Gisagara districts but moderate among children residing in Gatsibo, Kayonza, Rwamagana, Gasabo, Rulindo, Karongi, Ngororero, and Rubavu. However, the risks are lowest among those children living in Gakenke, Nyagatare, and Kirehe districts. The standard deviation and credible intervals maps higher uncertainty in various parts of the country, particularly in the districts of Rubavu, Kirehe, Nyagatare, Rusizi, Nyamasheke, Nyaruguru and Gisagara. In the case of anemia, the risks are higher among the children living in Rutsiro, Nyabihu, Rulindom, Ngoma, and Gisagara, followed by the districts of Rusizi, Huye, Nyanza, Ruhango, Ngororero, Gakenke, Gasabo, Nyarugenge, Kicukiro, and Kayonza. However, the risks for anemia are lower in the districts of Nyamasheke, Nyamagabe, Karongi, Muhanga, Kamonyi, Bugesera, Rwamagana, Gatsibo, Gicumbi, Nyagatare, Burera, Musanze and Rubavu. Nyaruguru and Kirehe are the districts with the lowest risks.

The nonlinear effects of child's age, age of household head, annual precipitation and mean temperature for the shared effects are presented in Figure 4 while those for the specific effects of malaria and anemia are presented in Figures 5 and 6 respectively. The Figures show the posterior means and 95% credible intervals. The findings show a negative relationship between the likelihood of suffering from both diseases and the age of the child, indicating that as a child gets older, the likelihood of suffering from both diseases decreases. The estimates for household head show higher likelihood among children whose head of household is 75 years or older. For precipitation, likelihood of shared risk increases with precipitation up to 120 mm/day, but starts decreasing when precipitation exceeds 120 mm/day though the credible intervals are generally higher. The findings revealed a positive association between temperature and risk of contracting both diseases, such that as temperature increases, the children are become more prone to the two illnesses.

The findings for malaria 5 reveal a positive association between likelihood of having malaria and child's age up to around 30 months but beyond this age, the likelihood decreases. Like for the shared pattern, the likelihood of suffering from malaria increases for children whose household head is older than 75, but lower before this age. The chances of having malaria increases with precipitation below 135 mm/day, and above this precipitation, the chances of having malaria decreases. As for temperature, the study findings reveal higher credible interval especially for the upper interval thus, the estimates may need to be interpreted with caution. The results for anemia, the non-linear effects of child's age, age of household head, annual precipitation and mean temperature are presented in Figure 6. The findings for anemia (Figure 6) show a negative association between the risk of having anemia and the child's age similar to what was obtained for the shared component, while in the case of age of household head, the risks are similar throughout the ages. Moreover, the risk of having anemia rises with increase in precipitation while the estimates for temperature appears flat but with wider upper credible interval.

Discussion

A shared component modeling approach was utilized to map co-morbidity of malaria and anaemia among under-five children in Rwanda. The Bayesian spatial model allows the mapping of spatial phenomena across small geographical units and to quantify

the effects of both socio-demographic and climatic factors on comorbidity of the diseases. The findings of study have practical relevance in a country like Rwanda where children still suffer co-morbidity from several diseases. Moreover, they provide evidence for effective and efficient implementation of intervention programs and policies designed to eliminate malaria and anaemia in Rwanda. The linear effects have shown the significant association between socio-demographic characteristics of the children and co-morbidity from the two diseases. The findings show no significant difference in the likelihood of suffering from both disease among under-five children residing in urban and rural areas. However, findings from some African countries have shown that under-five children from urban settings are less likely to suffer from both malaria and anaemia when compared with those from rural settings [22, 23], while a Rwandan study has demonstrated lower likelihood for malaria among urban children. In most rural settings in Rwanda, where more than 83 percent of the population reside, the majority of caregivers are farmers and small scale traders who possess limited access to information on disease control and prevention methods when viewed in respect to the urban dwellers. Typical of most rural settings in the developing world, they also suffer from shortage of health professionals and healthcare facilities and thus, overstretching the available few [1].

The findings of the study reveal significant association between household wealth and the likelihood of comorbidity from malaria and anaemia indicating that as wealth increases, the chances of co-morbidity reduces. These findings are consistent with what [24] and [25] found that children from impoverished households have higher prevalence of malaria than those from wealthy families. This may be attributed to lack of adequate and balanced diet among children from poor families. Moreover, wealthy households would have adequate resources for preventive and curative measures that safe guide their children from diseases. The similar significant association between household wealth and likelihood of having malaria and anaemia among under-five children have been reported in several other places [2, 23, 26].

We found that bed nets ownership reduce the risk of co-morbidity from malaria and anaemia but this was not the case for usage. Regular use of bed nets especially insecticide treated nets is a major preventive measure against malaria and by extension, anemia [24]. A previous analysis of the data used in this study but where only malaria was consider revealed that both ownership and usage of bed nets reduce the risk of malaria [1]. The non-significant estimate obtained for usage in this case therefore indicates that the usage of bed nets is not sufficient to cause a difference in co-morbidity from both diseases as it does for only malaria. Similar to some previous studies, we found children from male-headed household to be less prone to co-morbidity from the two diseases [24, 25]. As with most sub-Saharan African countries the majority of Rwandan households are male-headed who often provide protective leads to members of the family than what can be obtained in households headed by female. We however found no substantial difference when the sex of the child was considered.

The findings show an association between mother's educational attainment and likelihood of co-morbidity from the diseases. Children whose mothers completed at least primary education are less likely to suffer from both diseases. This association may be linked with the fact that higher educational attainment lead to improved knowledge, perception, and practice about

Figure Click here to access/download;Figure;FIG10.tiff

strategies for preventing and treating diseases [27, 28]. Educated mothers would know where to find and how to expressed themselves to physicians such that their children get the best of treatments than the uneducated ones will do.

The study findings revealed spatial heterogeneity in the shared risks of malaria and anaemia, and also from the individual diseases among under-five children in Rwanda. It is interesting to note that the spatial pattern obtained for malaria is similar to that observed for the shared risks of the two diseases more than does the map obtained for anemia. This finding further reinforces the synergy between the epidemiology causal pathways of malaria and anemia, that ensures that anemia is often a complication that could arise from malaria [2]. Districts with substantial high risks include Rutsiro, Nyabihu, Ruhango, Gisagara, and Rusizi, and moderate risks were estimated for Rubavu, Karongi, Ngororero, Kayonza, Gatsibo, Rwamagana, Gasabo and Rulindo. The existence of Gishwati forest, in which many agricultural and farming activities take place, could pose the highest risk of both diseases in the districts of Rutsiro and Nyabihu [29]. Also, the higher risk of co-morbidity of these diseases in Rusizi district may be aggravated by the abundance of breeding sites for anopheles mosquitoes in the Nyungwe forest and Bugarama plain [30]. The highest temperature in the Bugarama plain, a rice farming environment could also increase the risk of malaria transmission by mosquitoes [31]. The presence of several agricultural activities in Ruhango and Gisagara, coupled with cross border movement can define the increased likelihood of co-morbidity of malaria and anaemia. The irrigationbased agricultural practices mainly in the wetlands of the Eastern and Southern parts has been reported to influence malaria through the creation of vector breeding sites [32]. The spatial variation may be also linked with numerous factors including spatial disparity in human behaviour, awareness, and media exposure to malaria, child feeding practices, and disease prevention strategies. The high prevalence of malaria across the western provinces can be further explained by the presence of Lake Kivu which has been reported to speeds up malaria transmission [33].

The findings established nonlinear relationships between the shared risk of the two diseases and child's age, age of household head, annual precipitation, and mean temperature, which were considered as metrical covariates. The findings show high likelihood for the shared risks of malaria and anemia among children below 24 months of age, and this is similar to what was obtained for the specific component of anemia. Findings from other African countries have similarly exhibited this relationship [4]. The first two years of life is a period of intense growth and development which comes with high demand for iron, but which may not be sufficiently available especially for children from low socio-economic households. Weaning food may also be sourced from some unhygienic sources which could aggravate the risk of illness and subjecting the child to anemia. Moreover, due to the fragile nature of these children, they are highly susceptible to infections.

Temperature was revealed to be positively associated with the shared risks of malaria and anaemia. An increase in temperature could reduce the period it takes for new generations of mosquitoes to emerge, as well as the parasite's incubation period in mosquitoes [34]. Using an ecological model with malaria transmission data across African countries, [35] revealed that malaria transmission is at its optimal around temperature $25^\circ C$, but this decreases substantially when temperature exceeds $28^\circ C$. The

Figure Click here to access/download;Figure;FIG11.tiff

findings on annual precipitation reveals that when it is below 120 mm/day, the likelihood of co-morbidity of malaria and anemia increases with precipitation but when it is exceeds 120 mm/day, the risk decreases. Precipitation greater than 140mm destroys mosquitoes sites and leads to decrease in malaria transmission. The amount of precipitation determines the ample quantity and length of malaria transmission across different locations in a manner that in lower altitude regions, increase in precipitation might go with increase of available anopheles sites [36, 37].

Conclusion

This study aimed at understanding the shared spatial risks of malaria and anemia among under-five in Rwanda using a shared component model while controlling for other important determinants. The study reveals that socio-demographic characteristics such as place of residence, mother's educational level, wealth index, sex of household head, bed net ownership are among the major risk factors of the shared risk of the two diseases among Rwandan children. Rising precipitation particularly above 120 mm/day is associated with decreasing risk of co-morbidity of diseases. Furthermore, the risk of developing both diseases increases as the temperature rises. Findings from the study allow for the identification of the uneven spatial variations in joint and specific risks of suffering from malaria and anemia among young children in the country. Specifically, children from Rutsiro, Nyabihu, Ruhango, Rusizi, and Rusizi Districts are particularly at higher risks. The regional variation of malaria is nearly identical to the spatial pattern of the shared risk of both diseases, indicating that malaria could the leading cause of anemia among children in the country. The study recommends considerable efforts to improve the existing intervention programs and control strategies that prioritize higher risk regions. Moreover, a special attention to individuals living in lowest wealth quintiles, female headed households, and children of uneducated mothers should be considered when developing malaria and anemia intervention programs and policies. Also, the study recommends that when formulating malaria and anemia intervention programs, climate and environmental factors particularly temperature and precipitation should be taken into account.

Declarations

Ethics approval and consent to participate: Not applicable

Consent for publication: Not applicable

Availability of data and materials: The data are openly available upon request from The DHS Program (www.dhsprgram.com)

Figure

Click here to access/download;Figure;FIG12.tiff

Competing interests: The authors declare that they have no competing interests

Funding: The study received no specific funding

Authors’ contributions: Dr. Ezra Gayawan designed the study, provided the supervision and participated in drafting the manuscript. Pacifique Karekezi extracted the data, wrote the code, performed the analysis and Jean Damascene Nzabakiriraho participated in drafting the manuscript. All authors read and approved the final manuscript.

Acknowledgements: The authors acknowledge The DHS Program for granting access to the data.

Authors’information:Malaria and anemia are two of the most common diseases among Rwandan children under the age of five. By using a shared component model and adjusting for other important determinants, this study intended to identify the shared spatial risks of malaria and anemia among Rwandan children under the age of five. Children from Rutsiro, Nyabihu, Ruhango, Gisagara, and Rusizi had the highest risk of contracting both diseases, according to our findings. In Rutsiro, Nyabihu, Ruhango, Gisagara, and Rusizi, malaria is very common.The spatial distributions for malaria show a pattern that is similar to the shared spatial pattern for the two diseases, leading to the conclusion that malaria is the leading cause of anemia in Rwandan children under the age of five. The study also reveals that socio-demographic characteristics such as place of residence, wealth index, household sex, and bed net ownership are among the risk factors for contracting both diseases; additionally, the risk of developing both diseases increases as temperature rises, and precipitation above 120 mm/day is associated with a lower risk of disease co-morbidity.

review

ORIGINALITY REPORT

38%	36%	18%	7%
SIMILARITY INDEX	INTERNET SOURCES	PUBLICATIONS	STUDENT PAPERS

PRIMARY SOURCES

1	library.nexteinstein.org<br>Internet Source	21%
2	bmcpublichealth.biomedcentral.com<br>Internet Source	6%
3	Kai-Wen Li, Daiyu Fujiwara, Akihiro Haga, Huisheng Liu, Li-Sheng Geng. "Physical density estimations of single- and dual-energy CT using material-based forward projection algorithm: a simulation study", The British Journal of Radiology, 2021<br>Publication	2%
4	www.ncbi.nlm.nih.gov<br>Internet Source	1%
5	Submitted to University of Rwanda<br>Student Paper	1%

6	www.researchsquare.com<br>Internet Source	1 %
7	repository.i3l.ac.id<br>Internet Source	1 %
8	Glory Chidumwa, Innocent Maposa, Paul Kowal, Lisa K. Micklesfield, Lisa J. Ware. "Bivariate Joint Spatial Modeling to Identify Shared Risk Patterns of Hypertension and Diabetes in South Africa: Evidence from WHO SAGE South Africa Wave 2", International Journal of Environmental Research and Public Health, 2021<br>Publication	1 %
9	archpublichealth.biomedcentral.com<br>Internet Source	1 %
10	docksci.com<br>Internet Source	<1 %
11	Samson B. Adebayo, Ezra Gayawan. "A Bivariate Analysis of the Spatial Distributions of Stunting and Wasting Among Children Under-Five in Nigeria", Journal of Development Policy and Practice, 2021<br>Publication	<1 %

12	Osafu Augustine Egbon, Omodolapo Somo-Aina, Ezra Gayawan. "Spatial Weighted Analysis of Malnutrition Among Children in Nigeria: A Bayesian Approach", Statistics in Biosciences, 2021<br>Publication	<1 %
13	www.thelancet.com<br>Internet Source	<1 %
14	doaj.org<br>Internet Source	<1 %
15	Jean Damascene Nzabakiriraho, Ezra Gayawan. "Geostatistical modeling of malaria prevalence among under-five children in Rwanda", BMC Public Health, 2021<br>Publication	<1 %
16	Submitted to The African Institute for Mathematical Sciences<br>Student Paper	<1 %
17	maps.cancer.gov<br>Internet Source	<1 %
18	Submitted to Sultan Qaboos University<br>Student Paper	<1 %
19	www.qeios.com	

	Internet Source	<1%
20	www.rhsupplies.org<br>Internet Source	<1%
21	Bradley T. Kerridge, Phu Tran. "Husband/Partner Intoxication and Intimate Partner Violence Against Women in the Philippines", Asia Pacific Journal of Public Health, 2016<br>Publication	<1%
22	Kabiru K. Salami, Nneka L. Umego. "Household Environment and Malaria in Pregnancy in Ibadan, Nigeria", Health, 2018<br>Publication	<1%
23	www.researchgate.net<br>Internet Source	<1%
24	D. Cocchi. "Spatio-temporal disease mapping using INLA", Environmetrics, 09/2011<br>Publication	<1%

Exclude quotes On Exclude matches Off

Exclude bibliography On
