# Supplementary material for: Modeling the shared risks of malaria and anemia in Rwanda
Source: PLoS One. 2024 Apr 22;19(4):e0298259. doi: 10.1371/journal.pone.0298259 (PMC11034660; doi:10.1371/journal.pone.0298259)
Supplement: Attachment — Submitted filename: Responses to Reviewer report.pdf [file pone.0298259.s004.pdf]

**RESPONSES TO ACADEMIC EDITOR AND REVIEWERS' COMMENTS**

We thank the Academic Editor and Reviewers for reading through our manuscript and for the useful comments provided, which have helped us to improve on the quality of the manuscript. We provide below, our responses to the comments and hope that this version would meet the minimum publishable standard of PLOS ONE.

## Academic editor

Comment 1

Please ensure that your manuscript meets PLOS ONE's style requirements, including those for file naming.

This has been done

Comment 2

Please update your submission to use the PLOS LaTeX template.

This has been done

Comment 3

We note that you have indicated that data from this study are available upon request. PLOS only allows data to be available upon request if there are legal or ethical restrictions on sharing data publicly.

There is no legal or ethical restrictions on sharing data publicly, for this time the used dataset was uploaded as a supporting information.

Comment 4

We note that Figures 1-3 in your submission contain [map/satellite] images which may be copyrighted. All PLOS content is published under the Creative Commons Attribution License (CC BY 4.0), which means that the manuscript, images, and Supporting Information files will be freely available online, and any third party is permitted to access, download, copy, distribute, and use these materials in any way, even commercially, with proper attribution. For these reasons, we cannot publish previously copyrighted maps or satellite images created using proprietary data, such as Google software (Google Maps, Street View, and Earth).

The uploaded figures are our own figures produced using R program; they are not map or satellite images. Due to the fact that all submitted figures are made by ourselves, no copy right is required. You can double-check the tif figures we re-uploaded.

Comment 5

Please remove your figures from within your manuscript file, leaving only the individual TIFF/EPS image files, uploaded separately. These will be automatically included in the reviewers' PDF.

The figure were removed from the manuscript file and the tiff image files were uploaded separately.

## Reviewer reports:

**PONE-D-23-02111 Shared component modelling of malaria and anemia: An estimation of the shared demographic, spatial and climatic factors.**

## Reviewer 1

Comment 1

Plagiarism: Similarity shows 38 %. Authors were advised to decrease the percentage and go below 10 % at least.

The percentage has been decrease below 10%

Comment 2

Ethical clearance.

The study used a secondary data obtained from the Demographic and Health Survey. Data are available to anyone who wishes to conduct study, and after registering, it is permitted to utilize the data for research.

Comment 3

Can authors add a table of socio-economic factors for interviewees?

This has been done

Comment 4

The reasons behind the highest risks of contracting malaria and anemia that can be found among children from Rutsiro, Nyabihu, Ruhango, Gisagara and Rusizi districts, while those form Rubavu, Karongi, Ngororero, Kayonza, Gatsibo, Rwamagana, Gasabo and Rulindo have moderate risks of contracting both diseases.

This has been added into the manuscript.

## Reviewer 2

Comment 1

In the methods sections, kindly describe the outcome of interest and how comorbidity was generated. Also talk about the other predictors and any modifications you made, if any. You essentially need a subheading on the Dependent and independent variables.

This has been done

Comment 2

The DHS uses a complex survey design. Kindly describe how you accounted for complex survey design in the analysis, both univariate and spatial, if any.

This has been done

Comment 3

I am assuming the spatial shape files were used. If that was the case, kindly provide some details in the methods.

This has been done

Comment 4

If your approach to the spatial mapping used the geolocation data, then in the ethics section kindly discus how and why the displacement of the GPS locations of the centre points of the clusters was done in the DHS, and how this may affect the accuracy of the spatial maps ,if any

This has been done

Comment 5

Which software did you use to perform the demographics and spatial analysis? Please talk about the software used.

This has been done

Comment 6

Add a section on limitations of the study.

This has been done

Comment 7

In summary, kindly add more information in the methods so that the average reader can follow what was done step by step. Otherwise, great piece. I will look forward to the reading your revisions.

This has been done
