# Supplementary material for: Modeling the shared risks of malaria and anemia in Rwanda
Source: PLoS One. 2024 Apr 22;19(4):e0298259. doi: 10.1371/journal.pone.0298259 (PMC11034660; doi:10.1371/journal.pone.0298259)
Supplement: Attachment — Submitted filename: Review Report.docx [file pone.0298259.s002.docx]

**REVIEW REPORT OF A PAPER ENTITLED “Shared component modeling of malaria and anemia: An estimation of the shared demographic, spatial and climatic factors”**

**Comment 1:**  Plagiarism: Similarity shows 38% and a lot came from **library.nexteinstein.org** (21%). Authors are advised to decrease this percentage and go below 10% at least

**Comment 2:** Ethical Clearance: Authors should provide sufficient information on how ethical clearance although the used data are secondary data. Who has provided that ethical clearance? What about the ethical clearance certificate Number (as an example: we used one in 2020 with the number as follows: No 235/CMHS IRB/2020)? In Rwanda ethical clearance are normally given by the College of Medicine and Health Science [Institutional Review Board](https://www.sciencedirect.com/topics/engineering/institutional-review-board) at the University of Rwanda.

**Comment 3:** Can authors add a table of socio-economic factors for interviewees?

**Comment 4:**  What are the reasons behind this “**the highest risks of contracting malaria and anemia can be found among children from Rutsiro, Nyabihu, Ruhango, Gisagara, and Rusizi districts, while those from Rubavu, Karongi, Ngororero, Kayonza, Gatsibo, Rwamagana, Gasabo and Rulindo have moderate risks of contracting both diseases**”?

**N.B: I do expect to read this again deeply after decreasing the Similarity percentage**
